# Retraction: Genetic architecture modulates diet-induced hepatic mRNA and miRNA expression profiles in diversity outbred mice

**DOI:** 10.1093/genetics/iyab067

**Published:** 2021-07-14

Editor’s Note

This is a correction to: *GENETICS*, Volume 216, Issue 1, September 2019. Pages 241–259, https://doi.org/10.1534/genetics.120.303481.

The revised manuscript, entitled “Genetic Architecture Modulates Diet-Induced Hepatic mRNA and miRNA Expression Profiles in Diversity Outbred Mice,” by Que *et al.* has been submitted by the authors as a replacement for the originally published version of the manuscript.

Following publication, the authors became aware that 17 samples were incorrectly labeled. The IDs of 12 samples were corrected and 5 samples were removed. All analyses were recompleted, and the results remained robust except for the Fisher's exact test reported on p.251 in Table 4.

This version of the manuscript has been reviewed by members of the Editorial Board and is being republished after retraction of the original version of the manuscript.

